# Inequalities in urban greenness and epigenetic aging: Different associations by race and neighborhood socioeconomic status

**DOI:** 10.1126/sciadv.adf8140

**Published:** 2023-06-28

**Authors:** Kyeezu Kim, Brian T. Joyce, Drew R. Nannini, Yinan Zheng, Penny Gordon-Larsen, James M. Shikany, Donald M. Lloyd-Jones, Ming Hu, Mark J. Nieuwenhuijsen, Douglas E. Vaughan, Kai Zhang, Lifang Hou

**Affiliations:** ^1^ Department of Preventive Medicine, Northwestern University Feinberg School of Medicine, Chicago, IL, USA.; ^2^ Department of Nutrition, Gillings School of Global Public Health, University of North Carolina at Chapel Hill, Chapel Hill, NC, USA.; ^3^ Division of Preventive Medicine, Heersink School of Medicine, University of Alabama at Birmingham, Birmingham, AL, USA.; ^4^ School of Architecture, University of Notre Dame, Notre Dame, IN, USA.; ^5^ Institute for Global Health (ISGlobal), Barcelona, Spain.; ^6^ Department of Experimental and Health Sciences, Universitat Pompeu Fabra (UPF), Barcelona, Spain.; ^7^ CIBER Epidemiología y Salud Pública (CIBERESP), Madrid, Spain.; ^8^ Department of Medicine, Northwestern Feinberg School of Medicine, Chicago, IL, USA.; ^9^ Potocsnak Longevity Institute, Northwestern University Feinberg School of Medicine, Chicago, IL, USA.; ^10^ Department of Environmental Health Sciences, University of Albany, State University of New York, Rensselaer, NY, USA.

## Abstract

Slower epigenetic aging is associated with exposure to green space (greenness); however, the longitudinal relationship has not been well studied, particularly in minority groups. We investigated the association between 20-year exposure to greenness [Normalized Difference Vegetation Index (NDVI)] and epigenetic aging in a large, biracial (Black/white), U.S. urban cohort. Using generalized estimating equations adjusted for individual and neighborhood socioeconomic characteristics, greater greenness was associated with slower epigenetic aging. Black participants had less surrounding greenness and an attenuated association between greenness and epigenetic aging [β_NDVI5km_: −0.80, 95% confidence interval (CI): −4.75, 3.13 versus β_NDVI5km_: −3.03, 95% CI: −5.63, −0.43 in white participants]. Participants in disadvantaged neighborhoods showed a stronger association between greenness and epigenetic aging (β_NDVI5km_: −3.36, 95% CI: −6.65, −0.08 versus β_NDVI5km_: −1.57, 95% CI: −4.12, 0.96 in less disadvantaged). In conclusion, we found a relationship between greenness and slower epigenetic aging, and different associations by social determinants of health such as race and neighborhood socioeconomic status.

## Teaser

Urban greenness is associated with biological aging, and inequality in greenness exposure may cause disparities in healthy aging.

## INTRODUCTION

More than half of the world’s population now lives in urban areas, and it is projected that around 68% will live in urban areas by 2050 (*1*). Urban green space (greenness) including parks, green roofs, and community gardens provides critical ecosystem services, and their potential benefits to healthy aging (including better cardiovascular health and lower mortality) were documented in the literature (*2*–*5*). Although potential pathways, including physical activity and social network and interaction, have been suggested to partially explain how surrounding greenness might affect health outcomes (*6*–*8*), the underlying molecular biological mechanisms of these associations remain unclear.

One possible mechanism is epigenetic modifications, such as aberrant DNA methylation levels, which are associated with both environmental exposures and health conditions. Accumulated exposures to environmental factors can stimulate DNA hyper- or hypomethylation over time to affect human health (*9*, *10*). Epigenome-wide association studies have identified residential greenness-associated differently methylated regions that showed enrichments in physical activity– and allostatic load–related biomarkers, mental health, metabolic disease, and neoplasms (*11*, *12*). A summary biomarker, DNA methylation–based biological age (epigenetic age), has been proposed as a predictive marker of age-related health outcomes. This is supported by multiple previous studies of epigenetic age that observed associations with cardiovascular disease (CVD), cancer, and mortality (*13*–*15*) as well as various health-related lifestyle and exposure variables (*16*). Exposure to greenness has been rarely linked with epigenetic age except for one cross-sectional study (*17*). Furthermore, no studies have examined the role of race and sex in the association between greenness and epigenetic age that are important to understand and reduce disparities in greenness exposure and its benefits (*18*, *19*). Here, we conducted the first longitudinal study to examine the associations between long-term greenness exposures and epigenetic age and then evaluate race and sex differences as well as effect modification by neighborhood deprivation.

## RESULTS

### Characteristics of study participants

Table 1 shows the characteristics of study participants at Year 20 (Y20) (2005–2006). Among 924 participants (mean age = 45.3 years, SD = 3.5 years) consisting of 376 Black and 548 white participants, 453 were men and 471 were women. Five hundred and four (54.5%) participants had parks within 5 km of their residential address. The mean Normalized Difference Vegetation Index (NDVI) value within a 5-km buffer radius 1 year before the Y20 visit was 0.38 (SD = 0.11). We observed that participants having parks within 5 km had lower NDVI value (mean: 0.35, SD: 0.09 for participants with parks; mean: 0.41, SD: 0.12 for those without parks, respectively). The correlations of epigenetic age acceleration (EAA) measurements were moderate, ranged from 0.33 to 0.60 (table S1).

**Table 1.** Participants’ demographic characteristics at Coronary Artery Risk Development in Young Adults Exam Year 20 (2005–2006; *N* = 924). The NDVI values in the table represent the maximum values of NDVI measured 1-, 2-, and 3-year(s) pre-exam at Y20 (2005–2006).

Characteristic		
Age, mean (SD*)		45.3 (3.5)
Sex, *N* (%)	Men	453 (49.0)
	Women	471 (51.0)
Self-reported race, *N* (%)	Black participants	376 (40.7)
	White participants	548 (59.3)
Years of education, mean (SD)		15.1 (2.5)
Marital status, *N* (%)	Married	536 (58.0)
	Not married	388 (42.0)
Annual household income, *N* (%)	Less than $35,000	191 (20.7)
	$35,000–$74,999	270 (29.2)
	Greater than $75 000	463 (50.1)
Body mass index, mean (SD)		29.2 (6.4)
Physical activity (total intensity scores), mean (SD)		346.0 (274.4)
Smoking status, *N* (%)	Never	555 (60.1)
	Former	191 (20.7)
	Current	178 (19.3)
Neighborhood deprivation score, median (IQR^†^)		−0.47 (1.43)
Distance to the nearest major park (km), mean (SD)		6.94 (7.64)
Having parks within 5 km, *N* (%)	Yes	505 (54.6)
	No	419 (45.4)
NDVI^‡^ 5-km buffer (1 year before exam), mean (SD)		0.38 (0.11)
NDVI 5-km buffer (2 years before exam), mean (SD)		0.38 (0.11)
NDVI 5-km buffer (3 years before exam), mean (SD)		0.37 (0.11)
Study field center, *N* (%)	Birmingham, AL	216 (23.4)
	Chicago, IL	199 (21.5)
	Minneapolis, MN	251 (27.2)
	Oakland, CA	258 (27.9)

*SD, standard deviation.

†IQR, interquartile range.

‡NDVI, Normalized Difference Vegetation Index.

The distributions of having parks and NDVI values by subgroups are presented in Table 2. In general, Black participants were likely to have less surrounding greenness compared to white participants (having parks within 5 km: 49.2% for Black participants and 58.4% for white participants, *P* value: 0.005; mean NDVI_5km_: 0.36 for Black participants and 0.38 for white participants, *P* value: 0.007, respectively). The participants with higher neighborhood deprivation scores tended to have less greenness compared to those with lower scores (having parks within 5 km: 65.7% for below median and 40.3% for above median, *P* value <0.001). The distributions of having parks and NDVI values by field center are presented in table S2.

**Table 2.** Distribution of greenness variables (2005–2006; Y20) by race, sex, and neighborhood subgroups.

	Having parks within 5 km, yes; *N* (%)	*P value	NDVI 5-km buffer; mean (SD)	*P value
By race				
Black participants	185 (49.2)	0.005	0.36 (0.10)	0.007
White participants	320 (58.4)		0.38 (0.12)	
By sex				
Men	246 (54.3)	0.834	0.38 (0.11)	0.358
Women	259 (55.0)		0.37 (0.11)	
By neighborhood deprivation scores				
Below median	343 (65.7)	<0.001	0.38 (0.11)	0.180
Above median	162 (40.3)		0.37 (0.12)	

*P values for having parks and NDVI were derived from the chi-squared tests and Student’s *t* tests, respectively.

### Twenty-year exposure to greenness and epigenetic aging

**Fig. 1.**
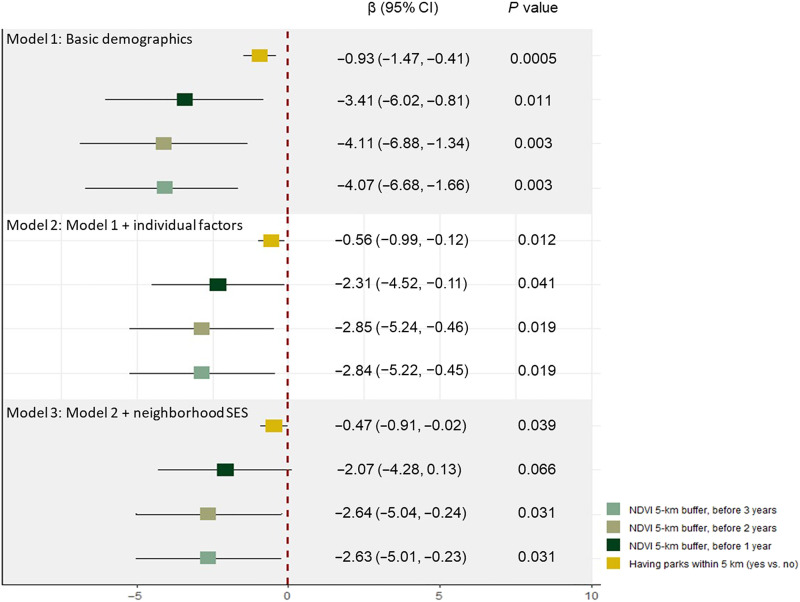
Association between long-term exposure to surrounding greenness (1985–2006; Y0-Y20) and GrimAA (2005–2006; Y20). Model 1: adjusted for age, sex, race, and study field center; model 2: adjusted for model 1 covariates + individual behavior and SES [education years, smoking, marital status, income, physical activity, and body mass index (BMI)]; model 3: adjusted for model 2 covariates + neighborhood deprivation score.

In Coronary Artery Risk Development in Young Adults (CARDIA) participants, having parks and greater exposure to residential-surrounding greenness from Y0 through Y20 (1985–2006) were associated with slower GrimAge acceleration (GrimAA) in the minimally adjusted model with attenuated results in models controlling for individual factors and particularly neighborhood socioeconomic status (SES) (Fig. 1). Compared to having no parks within 5 km of one’s residential address, having parks was associated with slower GrimAA of on average 0.47 to 0.93 years [β: −0.93, 95% CI: −1.47, −0.41 (model 1) to β: −0.47, 95% CI: −0.91, −0.02 (model 3)]. Measured 1 year before exam, each 0.1-unit higher NDVI_5km_ was associated with lower GrimAA of on average 2.07 to 3.41 years [β: −3.41, 95% CI: −6.02, −0.80) (model 1) to β: −2.07, 95% CI: −4.28, 0.13 (model 3)]. We observed similar patterns with NDVI_5km_ measured at different time points (2 and 3 years before exam), but the associations with NDVI measured 2 and 3 years before exam were generally even stronger than those for NDVI measured 1 year before exam. The longitudinal associations between surrounding greenness and GrimAA at Y15 were qualitatively similar to the results with GrimAA at Y20 (fig. S1).

**Fig. 2.**
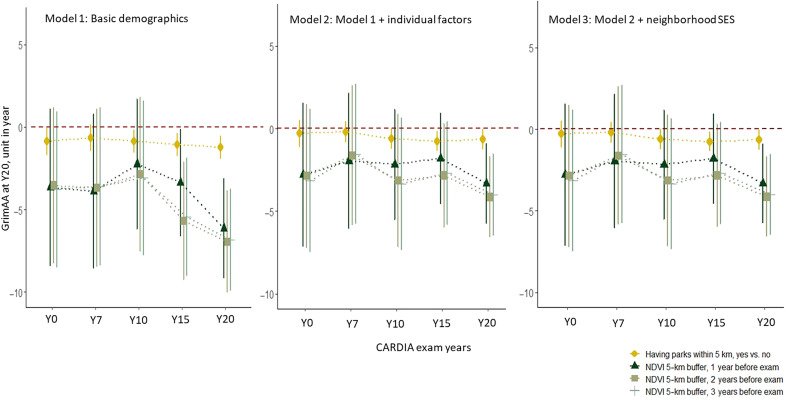
Time-specific associations between residential greenness at each exam visit and GrimAA at Y20 (2005–2006). Model 1: adjusted for age, sex, race, and field center; model 2: adjusted for model 1 covariates + individual behavior and SES (education years, smoking, marital status, income, physical activity, and BMI); model 3: adjusted for model 2 covariates + neighborhood deprivation score.

Figure 2 displays the time-specific association between residential greenness at each exam (Y0, Y7, Y10, Y15, and Y20) and GrimAA at Y20. Similar to the results from the associations with long-term exposure (Fig. 1), higher exposure to greenness was associated with slower GrimAA. We observed qualitatively consistent associations between greenness exposure at different time points and slower GrimAA in midlife, the strongest associations being those with greenness at Y20.

### Racial and SES disparities in greenness-epigenetic aging associations

**Fig. 3.**
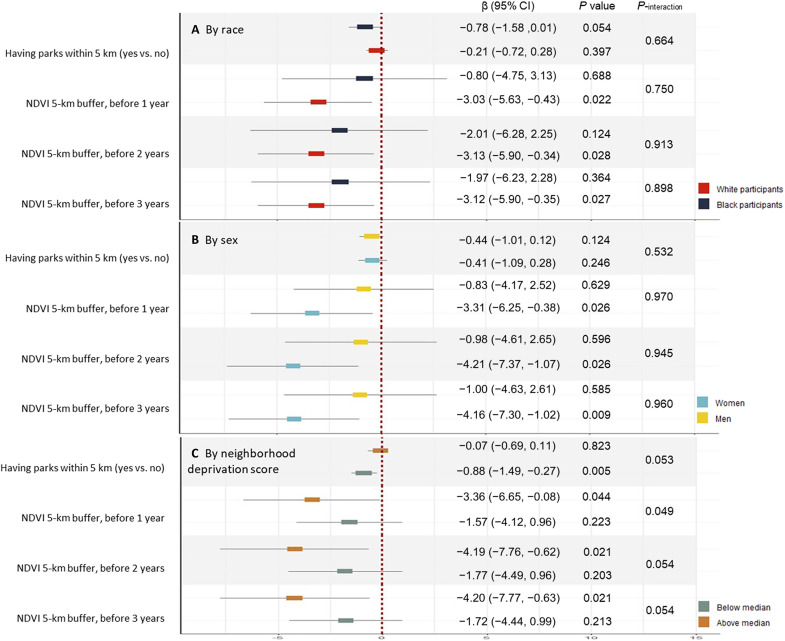
Association between long-term exposure to surrounding greenness (1985–2006; Y0-Y20) and GrimAA (2005–2006; Y20) by subgroups. Models were adjusted for age, sex, race, education years, smoking, marital status, income, physical activity, BMI, neighborhood deprivation score, and field center.

Figure 3 represents the results from our subgroup analyses. A marginal association between parks and GrimAA was observed among Black participants [β: −0.78, (95% CI: −1.58, 0.01)]. We observed associations of the NDVI_5km_ (1 year before exam) with GrimAA among white participants, showing on average 3.03 years slower GrimAA per 0.1 increment of NDVI [β: −3.03 (95% CI: −5.63, −0.43)] per 0.1 increment of NDVI. The magnitude of association was lesser among Black participants, showing 0.80 years slower GrimAA per 0.1 increment of NDVI [β: −0.80 (95% CI: −4.75, 3.13)]. We found associations of NDVI_5km_ (1 year before exam) with GrimAA among women [β: −3.31 (95% CI: −6.25, −0.38)] but not in men [β: −0.83 (95% CI: −4.17, 2.52)]. The associations between having parks and GrimAA were stronger among participants with lower deprivation scores showing, on average, 0.88 years slower GrimAA [β: −0.88 (95% CI: −1.49, 0.27)] among participants with lower scores and 0.07 years slower GrimAA [β: −0.07 (95% CI: −0.69, 0.11)] among participants with higher scores.

Conversely, the association of NDVI_5km_ (1 year after exam) was stronger among participants with higher deprivation scores, showing, on average, 3.36 years slower GrimAA [β: −3.36 (95% CI: −6.65, −0.08)] among participants with higher scores and 1.57 years slower GrimAA [β: −1.57 (95% CI: −4.12, 0.96)] among participants with lower scores per 0.1 increment of NDVI.

Among the individual components of GrimAA, longitudinal measures of NDVI 5-km buffer measured 3 years before the exam were inversely associated with DNA methylation surrogates for cystatin-C [β: −0.71 (95% CI: −1.39, −0.02)] and smoking pack-years [β: −0.57 (95% CI: −1.05, −0.08)]. We found no associations between the other components of GrimAA and residential surrounding greenness (fig. S2).

The associations between residential greenness and PhenoAge acceleration (PhenoAA) were qualitatively similar to the results with GrimAA but generally attenuated (tables S3 and S4). We did not observe any associations with DunedinPACE (tables S5 and S6).

### Results from sensitivity analyses

The results from our sensitivity analysis of the cross-sectional associations between various NDVI radial buffer sizes and GrimAA (table S7) were consistent with those from the main analysis for longitudinal associations, as expected as we observed high correlations among the various NDVI measures (*R*^2^ ranged from 0.89 to 0.99). The results from principal component (PC)-based GrimAA and PhenoAA (table S8) were almost identical to those from GrimAA and PhenoAA. The models with cumulative smoking and leukocyte compositions also showed generally consistent results with our main models (tables S9).

## DISCUSSION

To our knowledge, this is the first study to assess the associations between long-term exposure to surrounding greenness and epigenetic aging in a population-based cohort and how race and sex modify these associations. Longitudinally, having greater surrounding greenness was associated with slower epigenetic aging. The protective association of greenness with slower epigenetic aging was generally consistent across the study time points, suggesting that cumulative exposures to greenness may play a role in slower epigenetic aging later in life. In our data, Black participants tended to have less surrounding green space compared to the white participants, and beneficial association of greenness with epigenetic aging was only found among white participants. Our sex-stratified analyses exhibited protective associations of greenness in women but not in men. Greater exposure to greenness showed stronger associations among participants with higher neighborhood deprivation scores (i.e., more disadvantaged neighborhoods). We found most notable associations with GrimAA, and associations with PhenoAA were qualitatively similar to GrimAA results. We did not observe any associations with DunedinPACE. While much additional research remains to be done, in particular to identify explanatory factors and fully elucidate the relationship between SES and other social determinants of health, our findings provide a critical basis for potential biological mechanisms between greenness and improved health outcomes.

The protective association of greater exposure to greenness and slower epigenetic aging shown in our results is in line with prior evidence associating greenness exposure with improvements in health-related outcomes (*2*–*5*, *17*). In addition to the existing literature showing cross-sectional associations, we found that prolonged exposure to surrounding greenness from younger age was associated with slower epigenetic aging in midlife. Our observations were also supported by our time-specific analysis that displayed protective associations of greenness with slower GrimAA at Y20 consistently across the exam years. While the associations using prior-year greenness were qualitatively similar, the associations in more recent exam years were stronger. Coupled with our generalized estimating equation (GEE) models that showed less impact of greenness on GrimAA at Y15 than Y20, this could also imply that exposure to greenness could play an increasingly important role as people get older. Furthermore, the stronger associations using NDVI measured 2 and 3 years before exam compared to NDVI 1-year before exam in our results may suggest potential lag effects of surrounding greenness on changes in DNA methylation levels (i.e., years of exposure to greenness required for epigenetic effects). Future research is needed to more comprehensively identify the acting time frame, such as potential lag effects, in association with greenness exposure and DNA methylation. If confirmed, it could also emphasize the need for prompt intervention for the expansion of greenness in the urban area to promote slower epigenetic aging that can lead to better health outcomes in midlife.

Our subgroup analyses suggest that the expansion of greenness may be more important to the population with limited resources. We observed a weaker protective association of surrounding greenness with GrimAA among Black compared to white participants. In our data, Black participants had lesser surrounding greenness than white participants, suggesting that Black participants might have fewer opportunities for access to greenness that could result in less improvement in epigenetic aging. Together, our findings have implications that targeted urban strategies for greenness expansion may aid in improving environmental justice and health equity to overcome racial disparities in health. We also observed that women exhibited slower GrimAA with higher NDVI values, supporting prior evidence that showed a higher association of greenness with health among women (*20*). One explanation is that traditional social roles as a caregiver might increase the use of residential surrounding greenness (*20*, *21*). Another explanation is that the beneficial effects of surrounding greenness on health may be linked to the purpose of green space use, such as social networks and social support. Research indicates that the use of green space differs by sex; for example, women can visit parks more often potentially for social interaction and cohesion or as part of caring for children (*20*, *22*, *23*). In our study, residential surrounding greenness might serve as a place of restoration for social support and contribute to better health, as represented by slower epigenetic aging.

In our study, higher NDVI was associated with slower GrimAA among participants with higher deprivation scores. Our results with NDVI are in line with the studies that found stronger associations for greenness among participants with lower SES (*24*, *25*). Limited resources for leisure time activities have been suggested to explain the stronger associations of surrounding greenness among individuals with low SES, as it might increase the opportunity to be exposed to residential surrounding greenness (*19*). A study also showed that higher surrounding greenness area was associated with decreased stress levels in deprived neighborhoods (*26*). On the other hand, having parks within 5 km of one’s residential address was associated with slower GrimAA among participants with lower deprivation scores. The estimated protective effects of parks on GrimAA among less deprived participants in our study may be due to the different perceptions of park use between neighborhoods. It is possible that urban parks may be used for illicit activities (*27*, *28*), especially parks with low resources (*27*); the resulting crime and safety concerns may thus result in reduced park use by residents in low-income urban neighborhoods (*29*). Coupled with the lower NDVI among participants with parks within 5 km compared to those without in our data, the discrepancies between parks and NDVI by neighborhood SES suggest a need for future research that elucidates the role of other factors (such as actual use of parks, physical activity, and neighborhood characteristics) that may mediate the associations of parks and NDVI with epigenetic aging. Nevertheless, our results suggest that expansion of urban greenness with improved quality could contribute to slower epigenetic aging that may lead to better health outcomes, especially for people living in disadvantaged neighborhoods.

Long-term exposure to greenness was associated with two individual components of GrimAA (DNA surrogate cystatin-C and smoking pack-years) in our analyses, suggesting that exposure to greenness may modulate the epigenetic aging process via molecular processes. Elevated cystatin-C concentration is a useful clinical marker for poor kidney functions (*30*, *31*). Greenness-related factors, such as physical activity and air pollution, may play a role in the association between greenness and cystatin-C. Evidence suggests that physical activity is associated with lower concentrations of serum cystatin-C (*32*). Greenness is also known to have beneficial effects by mitigating the effects of air pollution (*33*), which, in the case of kidney function, include elevated cystatin-C concentrations (*34*, *35*). Meanwhile, smoking is an established risk factor for CVD and cancer, and research showed that DNA surrogate biomarkers of smoking were associated with EAA (*36*). Moreover, results from multiple studies imply inverse associations between neighborhood greenness and smoking (*37*, *38*). Some research suggests that living in areas with higher greenness may be associated with lower smoking uptake and higher smoking cessation (*37*, *39*). The underlying mechanisms between exposure to greenness and health are complex, and further study is needed to expand our understanding in this area. We believe that our results can contribute to the direction of the future study to incorporate molecular level changes, such as DNA methylation, to explore the acting mechanism of greenness exposure for health outcomes.

Our study showed qualitatively similar results between GrimAA and PhenoAA, suggesting protective associations of greenness exposure with slower epigenetic aging. However, we did not find associations with DunedinPACE. The differences in effect size between GrimAA and PhenoAA have been described in our previous study that showed associations of cumulative, collective lifestyle- and health-related exposures with epigenetic aging (*16*). Prior evidence has also proposed that different EAAs are linked to distinct physiological characteristics (*40*, *41*), and one study suggested GrimAA that has better prediction than PhenoAA of age-related physiologic characteristics and mortality (*41*). Recent studies comparing GrimAA and DunedinPACE observed different associations of both EAA measures with distinct health outcomes. A study showed associations of mortality with GrimAA but not with DunedinPACE (*42*). Another study found that cognitive decline was associated with DunedinPACE but not with GrimAA (*43*). The discrepancies in EAA associations with exposures and/or outcomes may be due to differences in the three EAA measurements’ component CpGs (*44*–*46*). The different CpG sets of these three EAAs may have different sensitivities to external stimuli (e.g., exposure to greenness) and lead to distinct associations with varying physiological characteristics by EAA measures as shown in other studies. Assessing the relationships across different types of exposures, EAA measurements, and health consequences should be one direction of future studies.

Our study is not without limitations. The measurements of surrounding greenness adopted in this study did not incorporate the quality of greenness or type of green spaces. More detailed greenness data may further elucidate the association between surrounding greenness and epigenetic aging. We also cannot rule out the possibility of residual confounding from factors not measured in this study, such as stress and social network. In addition, we did not address residential selectivity bias or other sources of bias related to the selection of residential locations on the basis of amenities like parks. Nor did we address biases related to the residential sorting of individuals of lower income into disadvantaged neighborhoods with lower access to parks. The observed attenuation of results in models adjusting for individual factors and neighborhood SES suggests that this may indeed be the case. We did not conduct a sensitivity analysis with and without movers; however, this cohort was launched in 1985, and only 7% of participants have stayed in their baseline addresses as of 2005–2006 (*47*). Furthermore, individuals’ social capital, including social networks and support, might play a role as a mediator in the association of greenness exposure and epigenetic aging by providing open space for social networking that may contribute to better health conditions (*48*, *49*). In sum, comprising quality and type of surrounding greenness as well as identifying the role of social capital and other potential mediators should be a direction of future studies to expand the understanding of the association between greenness and epigenetic aging. Last, additional studies in more cohorts with diverse populations across multiple areas will allow for better generalizability of the findings presented here.

In conclusion, the empirical findings from this study can provide scientific evidence to improve the exposure to greenness at a neighborhood level to modulate the epigenetic aging process by providing critical insights into the biological mechanisms between greenness and epigenetic aging. Our findings have strong implications for coupling public health intervention and urban planning to expand green infrastructure and maximize its utilization that may be associated with improved life span. The subgroup-specific associations presented in this study also contribute to the literature needed to support policy-level efforts to advance equity in green space in association with its health benefit. Future research exploring pathways with other explanatory factors including residential selection, individual park-use behavior, and neighborhood SES is needed for a more comprehensive understanding of greenness and epigenetic aging.

## MATERIALS AND METHODS

### Cohort description

Participants from the CARDIA Study were included in this investigation. CARDIA is a prospective cohort study that recruited participants between 1985 and 1986 (study baseline; Y0) from four urban field centers across the United States: Birmingham, AL; Chicago, IL; Minneapolis, MN; and Oakland, CA. A total of 5115 men and women (aged 18 to 30 years) consisting of Black and white individuals were recruited at baseline and received follow-up examinations at Y2 (1987–1988), Y5 (1990–1991), Y7 (1992–1993), Y10 (1995–1996), Y15 (2000–2001), Y20 (2005–2006), Y25 (2010–2011), Y30 (2015–2016), and Y35 (2021–2022). Further details of the CARDIA study are described elsewhere (*50*). Each CARDIA field center and the coordinating center received institutional review board approval from their respective institution, and all participants provided written informed consent at each exam.

The analytic cohort of this study comprised participants who returned for Y15 and Y20 exams, as DNA methylation profiling was conducted at these two exam points. Of 3672 and 3549 CARDIA participants who returned for Y15 and Y20 exams, respectively, we included 924 participants with complete information on DNA methylation levels, residential surrounding greenness, and other covariates at Y20.

### Estimation of residential surrounding greenness

In CARDIA, geocoding of participants’ residential addresses was performed at Y0, Y7, Y10, Y15, Y20, and Y25. On the basis of the geocodes, participants’ exposure to residential surrounding greenness was obtained through two measures: park exposure and surrounding greenness exposure from satellite-based estimation.

Park exposure (having parks within 5 km): The residential history was matched to the information on public parks based on the component of StreetMap Pro (version 5.2.) from the Environmental Systems Research Institute. The distance to the park was measured as a Euclidean distance (kilometers) to the major park nearest to each participant’s residential location. As a scatterplot between continuous measurement of the distance to the park and EAA did not display obvious trends (fig. S3), we derived a dichotomous variable using a threshold of 5 km (i.e., having parks within 5 km from participants’ residential address versus not) to be consistent with our main measurement of interest in NDVI analysis.

Surrounding greenness exposure (NDVI 5-km buffer): Surrounding greenness was estimated using the satellite-derived NDVI, which indicates the land surface’s overall greenness but not the greenness type (e.g., fields, forests and parks). We obtained NDVI data from the Global Inventory Modelling and Mapping Studies for Y0, Y7, and Y10 (before 2000) and Moderate Resolution Imaging Spectroradiometer for Y15 and Y20 (during 2000–2015). All NDVI data were downloaded from Earthdata Search of NASA (https://search.earthdata.nasa.gov/search). NDVI is calculated as a near-infrared–to–visible red ratio and ranges from −1 to 1, and we replaced negative values (for frozen ground, water, and nonvegetated soil) with zero to represent more greenness with a higher value of NDVI as a proportion (*51*). For example, an NDVI value of 0.2 with a 5-km buffer indicates that the greenness accounts for 20% of land surface within a radial distance of 5 km from a participant’s residential address (translates to 5 × 5 × 3.14 × 0.2 = 15.7 km^2^ of a green space area). In CARDIA, the 5-km buffer (NDVI_5km_) was available for all examination years included in this study (Y0, Y7, Y10, Y15, and Y20), but the other buffers (NDVI_250m_, NDVI_500m_, NDVI_1km_, and NDVI_2km_) were available only for Y20 as the high-resolution satellite images are rarely available publicly in early days; therefore, we used NDVI_5km_ as our primary measure of interest in main analyses, and other buffers at Y20 as measures in a sensitivity analysis for comparison purposes. To minimize the seasonal variation and examine potential lag effects, we used the maximum values of NDVI measured 1, 2, and 3 years before CARDIA exam visits.

### DNA methylation profiling and calculation of EAA

Participants’ DNA was extracted from whole blood among 1200 randomly selected participants at Y15 and Y20, and DNA methylation levels were measured via Infinium Methylation EPIC BeadChip (EPIC array). For quality control (QC) of the DNA methylation profiles, we included only CpGs with a detection rate greater than 95%. We used the R package ENmix (*52*) for QC procedures and the preprocessIllumina function in the minfi package for post-QC preprocessing procedures (*53*).

We included three DNA methylation-based epigenetic age measurements in this study: GrimAge, PhenoAge, and DunedinPACE. We calculated GrimAge and PhenoAge using Horvath’s online DNA Methylation Age Calculator (https://dnamage.genetics.ucla.edu) and DunedinPACE on the basis of the published algorithm (*44*–*46*). GrimAge was developed to predict time to death using data of 2356 individuals age in fifties to seventies from the Framingham Heart Study Offspring Cohort (predominantly white race, balanced sex). GrimAge incorporated chronological age, seven DNA surrogate biomarkers of plasma proteins (adrenomedullin, beta-2 microglobulin, cystatin C, growth differentiation factor 15, leptin, plasminogen activation inhibitor 1, and tissue inhibitor metalloproteinase 1), and smoking pack-years (*45*), which were associated with morbidity and mortality. PhenoAge was derived on the basis of the individuals age over 20 from the National Health and Nutrition Examination Survey III (*N* = 9926) and IV (*N* = 6209). PhenoAge incorporated chronological age and nine clinical biomarkers (albumin, creatinine, glucose, C-reactive protein, lymphocyte percent, mean cell volume, red cell distribution width, alkaline phosphatase, and white blood cell count) to predict “phenotypic age” (*44*). GrimAA represents the deviation of GrimAge from chronological age, calculated as the regression residuals of the GrimAge on chronological age therefore the values greater than zero represent accelerated epigenetic age in unit of year. PhenoAA, which represents the deviation of PhenoAge from chronological age, was calculated in the same way. DunedinPACE was developed on the basis of the data of 817 individuals age at 45 from the Dunedin Study (predominantly white race and balanced sex). DunedinPACE incorporated the longitudinal change of 19 biomarkers associated with clinical conditions including cardiovascular, metabolic, and immune systems to derive the individual differences in pace of aging which reflects age-related declines. The DunedinPACE values represent the rate of epigenetic age compared to the average rate in the same age group, and the values greater than 1 indicate accelerated epigenetic age (*46*).

### Covariates

We included the following covariates in the study: self-reported sex (men or women) and race (Black or white), years of education, body mass index (BMI), marital status, annual household income, physical activity, smoking status, neighborhood deprivation score, and study field center. Physical activity was measured using total intensity scores based on participants’ self-reported physical activity levels. Smoking status was classified into three groups: never, former, and current smoker. The neighborhood deprivation score adopted in this study was developed from a PC analysis of the four census tract-level indicators of SES: median household income, the proportion of the population at or below poverty level, the proportion of the population with less than high school education, and the proportion of the population with a college degree or higher education (*54*). The neighborhood deprivation score was designed to have values between −1 and 1, with higher values representing high socioeconomic disadvantage in participants’ neighborhoods. The score was matched to the participants’ geocoded residential location at each CARDIA exam visit.

### Statistical analysis

Participants’ characteristics at Y20 were presented as mean and SD for continuous variables [age, years of education, BMI (kg/m^2^), physical activity, distance to the park, and NDVI with different buffer sizes], median and interquartile range (IQR) for neighborhood deprivation score, and count and proportion (%) for categorical variables (race, sex, marital status, annual household income, smoking status, and field center). We examined the correlations among the EAA measurements and distributions of NDVI by status of having parks within 5 km. We also assessed the distributions of parks and NDVI by study field center.

To assess the association between 20-year exposure to residential surrounding greenness (independent variable) at Y0, Y7, Y10, Y15, and Y20 and EAA (dependent variable: GrimAA, PhenoAA, and DunedinPACE), at Y20, we adopted GEE regression models. We conducted separate analyses of different variables representing participants’ residential greenness: (i) having parks within 5 km, (ii) different NDVI buffer sizes measured 1, 2, and 3 years before the Y20 exam. In our main analysis, we used multiple models controlling for different sets of covariates. In model 1, we controlled for race, sex, and study field center. In model 2, we additionally controlled for individual SES and lifestyle factors (education, smoking, marital status, household income, physical activity, and BMI). Last, we added neighborhood SES (represented by neighborhood deprivation score) in model 3. We conducted analyses using GEE models with 15-year exposure to greenness (Y0, Y7, Y10, and Y15) EAA at Y15 for comparison. Notably, we treated the independent variables (greenness variables) and covariates (individual and neighborhood SES and lifestyle factors) as time-varying variables (Y0 to Y15 for Y15 analysis and Y0 to Y20 for Y20 analysis, respectively) while the dependent variable (EAA at Y15 and Y20) was fixed in the GEE models. As we found associations of greenness with GrimAA from the analyses with GEE models, we further performed analyses for time-specific associations with residential greenness at each exam (Y0, Y7, Y10, Y15, and Y20), and GrimAA at Y20 using linear regression models to investigate whether exposure at specific time points have greater associations with GrimAA.

We performed subgroup analyses to investigate the potential deviations in association of greenness with EAA by social determinants of health. For subgroup analyses, we performed analyses stratified by race, sex, and neighborhood deprivation score (dichotomized about the median). Race, sex, and neighborhood-specific interactions were tested by including a product term between greenness variables and each potential effect modifier in the full model with total participants. We also explored the associations between residential greenness and *Z*-score transformed eight individual DNA surrogate markers GrimAA to further investigate the potential biological mechanisms of exposure to greenness on GrimAA.

We conducted multiple sensitivity analyses to investigate the associations between residential surrounding greenness and EAA at Y20 by subgroups and various NDVI radial buffer sizes. We assessed the cross-sectional associations between residential greenness at Y20 and EAA at Y20 using different radial buffers: NDVI250m, NDVI500m, NDVI1km, NDVI2km, and NDVI5km. We investigated the associations of greenness with PCs-incorporated GrimAA and PhenoAA (PC-GrimAA and PC-PhenoAA, respectively), which incorporate the PCs from the CpGs to train the epigenetic age measurements, for comparison purposes (*55*). We assessed the associations of greenness with EAA additionally adjusting for cumulative smoking (smoking pack per year) and estimated leukocyte composition. The set of covariates in the primary analysis model 3 was used for all sensitivity analyses. We used SAS version 9.4 (SAS Institute Inc., Cary, NC) for all statistical analyses for this study.
