# Supplementary material for: Inequalities in urban greenness and epigenetic aging: Different associations by race and neighborhood socioeconomic status
Source: Sci Adv. 2023 Jun 28;9(26):eadf8140. doi: 10.1126/sciadv.adf8140 (PMC10306284; doi:10.1126/sciadv.adf8140)
Supplement: Supplementary file 1 — Figs. S1 to S3 Tables S1 to S9 [file sciadv.adf8140_sm.pdf]

Science Advances
AAAS

Supplementary Materials for

Inequalities in urban greenness and epigenetic aging: Different associations by race and neighborhood socioeconomic status

Kyeezu Kim *et al.*

Corresponding author: Kai Zhang, kzhang9@albany.edu; Lifang Hou, l-hou@northwestern.edu

Sci. Adv. **9**, eadf8140 (2023)
DOI: 10.1126/sciadv.adf8140

This PDF file includes:

Figs. S1 to S3
Tables S1 to S9

Supplementary Figure 1. Association between long-term exposure to surrounding greenness (1985-2001; Y0-Y15) and GrimAA (2000-2001; Y15, N=860).

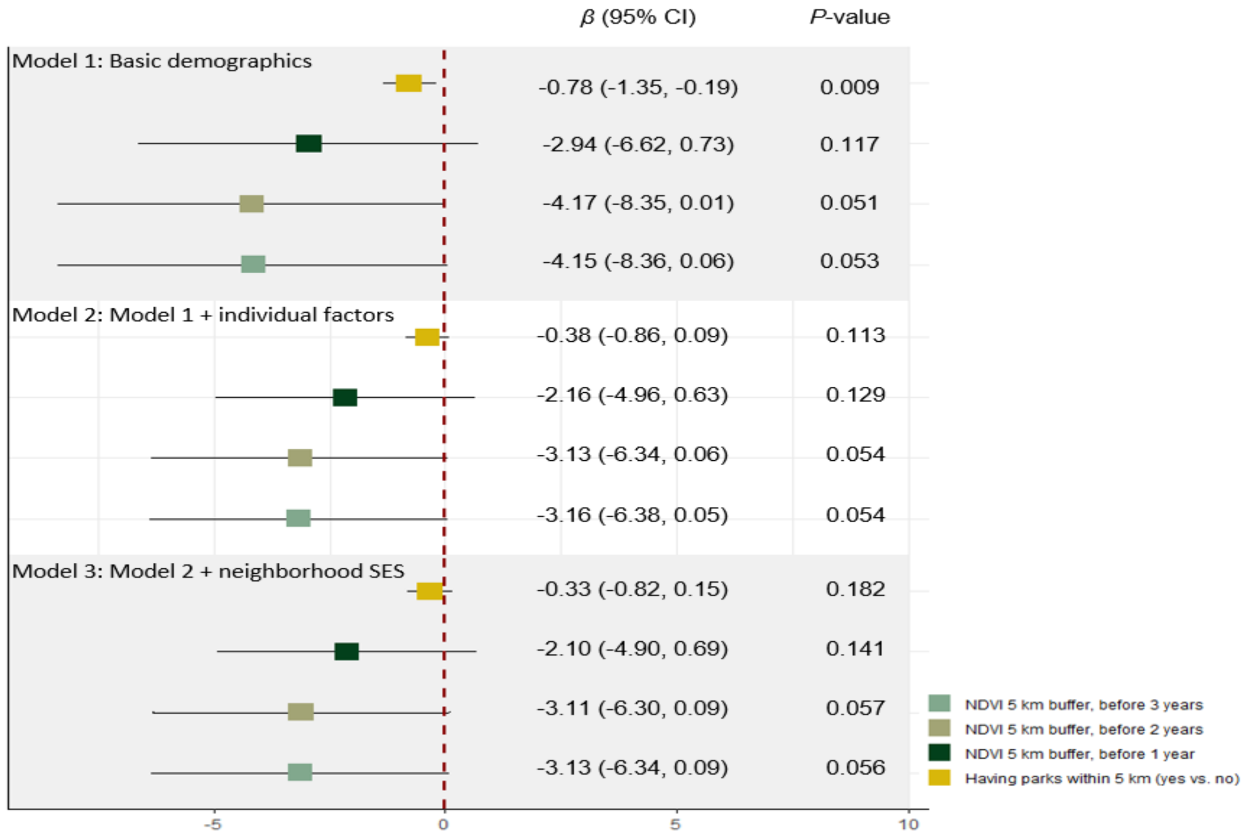

NDVI: Normalized Difference Vegetation Index. β for NDVI represents changes in GrimAA per 0.1 increment of NDVI.

Model 1: adjusted for age, sex, race, and field center; Model 2: adjusted for Model 1 covariates + individual behavior and SES (education years, smoking, marital status, income, physical activity, and BMI); Model 3: adjusted for Model 2 covariates + neighborhood deprivation score.

Supplementary figure 2. Association between long-term exposure to residential greenness (1985-2006; Y0-Y20) and DNA surrogate markers of individual components of GrimAA (2005-2006; Y20).

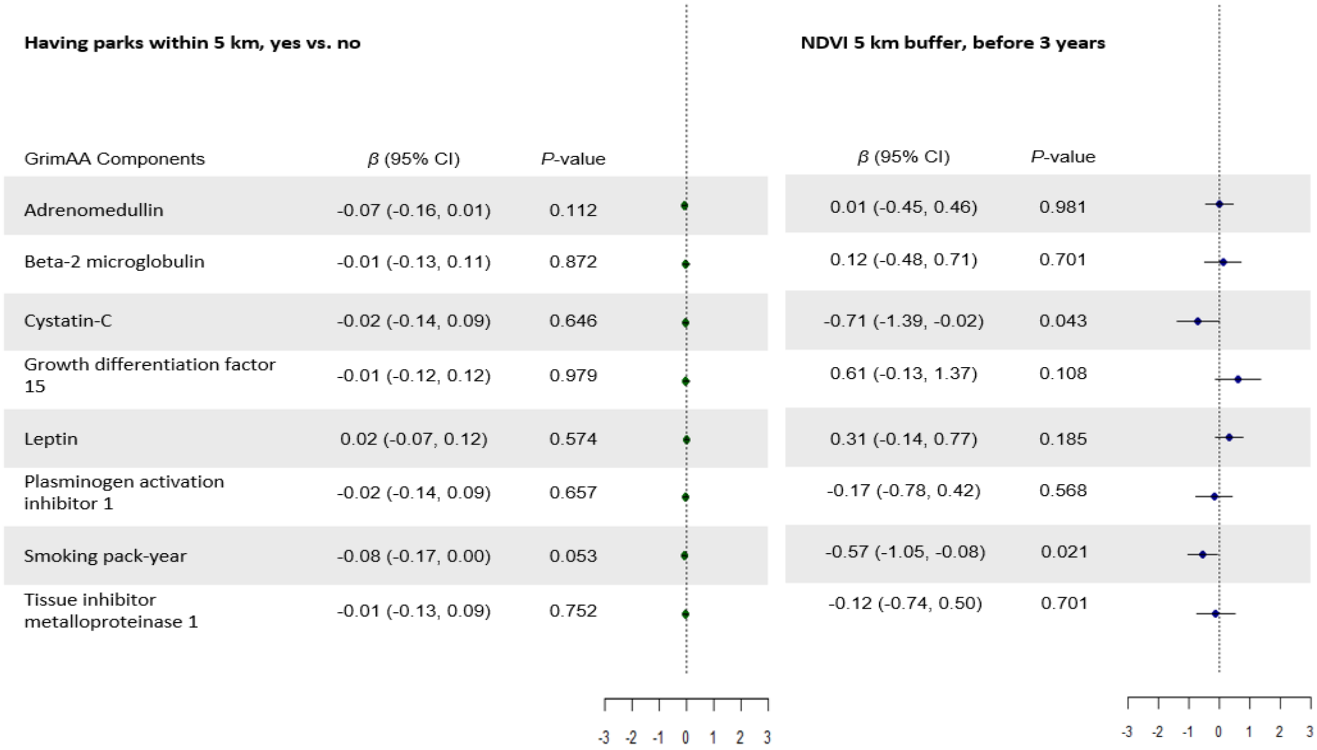

NDVI: Normalized Difference Vegetation Index.
Models were adjusted for age, sex, race, education years, smoking, marital status, income, physical activity, BMI, neighborhood deprivation score, and study center.

Supplementary Figure 3. Scatterplot of distance to the nearest major park and GrimAA at Y20 (2005-2006).

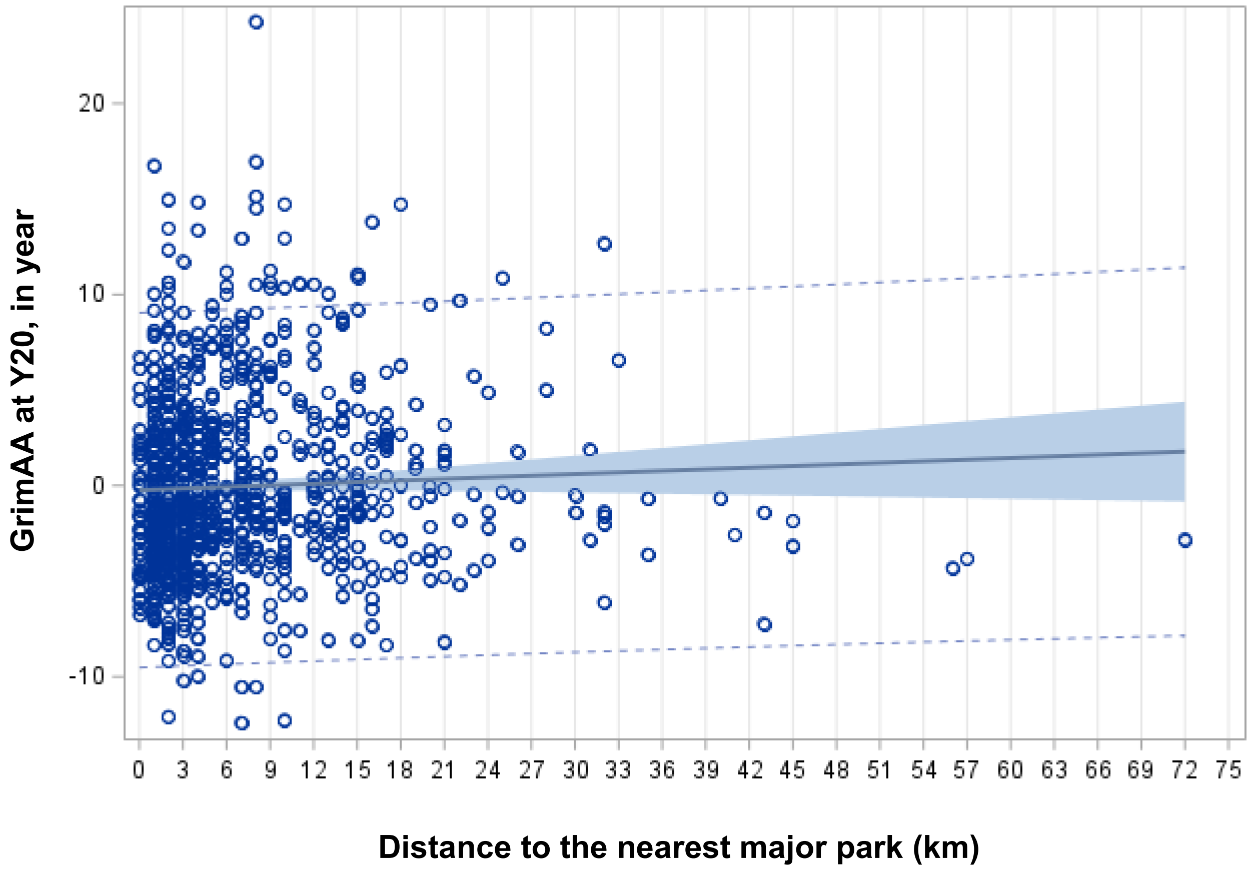

Supplementary Table 1. Pearson's correlations of EAA measurements at Y20

	GrimAA	PhenoAA	DunedinPACE
GrimAA	1.00	0.33 (P<.001)	0.60 (P<.001)
PhenoAA		1.00	0.35 (P<.001)
DunedinPACE			1.00

Supplementary Table 2. Distributions of greenness variables at Y20 by field center

	Having parks within 5km, yes N (%)	*P*-value	NDVI 5km buffer mean (SD)	*P*-value
Birmingham, AL	31 (14.3)	<.001	0.52 (0.07)	<.001
Chicago, IL	102 (51.3)		0.30 (0.08)	
Minneapolis, MN	186 (74.1)		0.36 (0.06)	
Oakland, CA	186 (72.1)		0.34 (0.10)	

P-values for having parks and NDVI were derived from Chi-squared test and ANOVA test, respectively.

Supplementary Table 3. Association between long-term exposure to residential surrounding greenness (1985-2006; Y0-Y20) and PhenoAA (2005-2006; Y20).

Greenness variables	Model 1: Basic demographics		Model 2: Model 1 + individual factors		Model 3: Model 2 + neighborhood SES	
	β (95% CI)	*P*-value	β (95% CI)	*P*-value	β (95% CI)	*P*-value
Having parks within 5 km, yes vs. no	-0.50 (-1.25, 0.24)	0.184	-0.29 (-1.02, 0.44)	0.432	-0.28 (-1.01, 0.45)	0.455
NDVI 5km buffer (1yr before exam)	-3.15 (-7.48, 1.17)	0.153	-2.73 (-7.17, 1.71)	0.228	-2.65 (-7.09, 1.79)	0.242
NDVI 5km buffer (2yr before exam)	-4.13 (-8.66, 0.40)	0.074	-3.76 (-8.44, 0.91)	0.115	-3.72 (-8.39, 0.95)	0.118
NDVI 5km buffer (3yr before exam)	-4.13 (-8.66, 0.41)	0.074	-3.77 (-8.44, 0.90)	0.113	-3.73 (-8.39, 0.94)	0.117

NDVI: Normalized Difference Vegetation Index. β for NDVI represents changes in PhenoAA per 0.1 increment of NDVI.
Model 1: adjusted for age, sex, race, and field center; Model 2: adjusted for Model 1 covariates + individual behavior and SES (education years, smoking, marital status, income, physical activity, and BMI); Model 3: adjusted for Model 2 covariates + neighborhood deprivation score.

Supplementary Table 4. Association between long-term exposure to residential surrounding greenness (1985-2006; Y0-Y20) and PhenoAA (2005-2006; Y20) by subgroups.

Surrounding greenness	β (95% CI)	*P*-value	β (95% CI)	*P*-value
By race	Black participants (N=376)		White participants (N=548)	
Having parks within 5 km, yes vs. no	0.70 (-0.43, 1.84)	0.227	-0.41 (-1.34, 0.51)	0.384
NDVI 5km buffer (1yr before exam)	-0.81 (-8.50, 6.87)	0.836	-3.39 (-8.83, 2.05)	0.222
NDVI 5km buffer (2yr before exam)	-1.54 (-9.71, 6.64)	0.713	-4.52 (-10.20, 1.16)	0.119
NDVI 5km buffer (3yr before exam)	-1.65 (-9.84, 6.54)	0.693	-4.48 (-10.14, 1.18)	0.121
By sex	Men (N=453)		Women (N=471)	
Having parks within 5 km, yes vs. no	-0.47 (-1.41, 0.45)	0.316	0.01 (-1.10, 1.12)	0.986
NDVI 5km buffer (1yr before exam)	-0.86 (-7.29, 5.56)	0.792	-4.56 (-10.49, 1.36)	0.131
NDVI 5km buffer (2yr before exam)	-1.05 (-7.77, 5.68)	0.760	-6.32 (-12.55, -0.09)	0.047
NDVI 5km buffer (3yr before exam)	-1.07 (-7.77, 5.63)	0.754	-6.32 (-12.54, -0.09)	0.047
By neighborhood SES	Below median (N=522)		Above median (N=402)	
Having parks within 5 km, yes vs. no	-0.59 (-1.59, 0.41)	0.248	0.51 (-0.52, 1.53)	0.337
NDVI 5km buffer (1yr before exam)	-2.08 (-7.70, 3.54)	0.468	-3.12 (-9.18, 2.94)	0.313
NDVI 5km buffer (2yr before exam)	-2.55 (-8.43, 3.33)	0.396	-5.08 (-11.37, 1.21)	0.114
NDVI 5km buffer (3yr before exam)	-2.47 (-8.32, 3.38)	0.408	-5.21 (-11.56, 1.13)	0.107

NDVI: Normalized Difference Vegetation Index. β for NDVI represents changes in PhenoAA per 0.1 increment of NDVI. Models were adjusted for age, sex, race, education years, smoking, marital status, income, physical activity, BMI, neighborhood deprivation score, and field center.

Supplementary Table 5. Association between long-term exposure to residential surrounding greenness (1985-2006; Y0-Y20) and DunedinPACE (2005-2006; Y20).

Greenness variables	Model 1: Basic demographics		Model 2: Model 1 + individual factors		Model 3: Model 2 + neighborhood SES	
	β (95% CI)	*P*-value	β (95% CI)	*P*-value	β (95% CI)	*P*-value
Having parks within 5 km, yes vs. no	-0.01 (-0.02, 0.00)	0.072	-0.00 (-0.01, 0.01)	0.468	-0.00 (-0.01, 0.01)	0.791
NDVI 5km buffer (1yr before exam)	-0.04 (-0.11, 0.03)	0.292	0.02 (-0.05, 0.08)	0.596	0.01 (-0.05, 0.09)	0.629
NDVI 5km buffer (2yr before exam)	-0.05 (-0.13, 0.02)	0.144	0.00 (-0.06, 0.07)	0.936	-0.00 (-0.08, 0.07)	0.930
NDVI 5km buffer (3yr before exam)	-0.05 (-0.13, 0.02)	0.162	0.00 (-0.01, 0.07)	0.894	-0.00 (-0.08, 0.07)	0.961

NDVI: Normalized Difference Vegetation Index. β for NDVI represents changes in DunedinPACE per 0.1 increment of NDVI.
Model 1: adjusted for age, sex, race, and field center; Model 2: adjusted for Model 1 covariates + individual behavior and SES (education years, smoking, marital status, income, physical activity, and BMI); Model 3: adjusted for Model 2 covariates + neighborhood deprivation score.

Supplementary Table 6. Association between long-term exposure to residential surrounding greenness (1985-2006; Y0-Y20) and DunedinPACE (2005-2006; Y20) by subgroups

Surrounding greenness	β (95% CI)	*P*-value	β (95% CI)	*P*-value
By race	Black participants (N=376)		White participants (N=548)	
Having parks within 5 km, yes vs. no	-0.01 (-0.03, 0.00)	0.227	0.01 (-0.01, 0.02)	0.308
NDVI 5km buffer (1yr before exam)	0.03 (-0.08, 0.15)	0.603	0.01 (-0.07, 0.09)	0.836
NDVI 5km buffer (2yr before exam)	-0.01 (-0.14, 0.11)	0.790	0.01 (-0.07, 0.10)	0.765
NDVI 5km buffer (3yr before exam)	-0.01 (-0.13, 0.11)	0.829	0.01 (-0.07, 0.10)	0.751
By sex	Men (N=453)		Women (N=471)	
Having parks within 5 km, yes vs. no	-0.01 (-0.03, 0.00)	0.075	0.01 (-0.01, 0.03)	0.156
NDVI 5km buffer (1yr before exam)	0.06 (-0.03, 0.15)	0.198	-0.01 (-0.11, 0.07)	0.690
NDVI 5km buffer (2yr before exam)	0.05 (-0.03, 0.15)	0.243	-0.04 (-0.14, 0.05)	0.382
NDVI 5km buffer (3yr before exam)	0.05 (-0.03, 0.15)	0.235	-0.04 (-0.14, 0.05)	0.406
By neighborhood SES	Below median (N=522)		Above median (N=402)	
Having parks within 5 km, yes vs. no	-0.00 (-0.01, 0.01)	0.748	0.00 (-0.01, 0.01)	0.937
NDVI 5km buffer (1yr before exam)	-0.00 (-0.08, 0.08)	0.979	0.04 (-0.04, 0.14)	0.334
NDVI 5km buffer (2yr before exam)	-0.01 (-0.09, 0.07)	0.869	0.02 (-0.08, 0.12)	0.676
NDVI 5km buffer (3yr before exam)	-0.00 (-0.08, 0.08)	0.913	0.02 (-0.08, 0.12)	0.653

NDVI: Normalized Difference Vegetation Index. β for NDVI represents changes in DunedinPACE per 0.1 increment of NDVI. Models were adjusted for age, sex, race, education years, smoking, marital status, income, physical activity, BMI, neighborhood deprivation score, and field center.

Supplementary Table 7. Sensitivity analysis stratified by residential surrounding greenness (Y20) and GrimAA (Y20) using different NDVI buffer sizes.

	Model 1: Basic demographics		Model 2: Model 1 + individual factors		Model 3: Model 2 + neighborhood SES	
	β (95% CI)	*P*-value	β (95% CI)	*P*-value	β (95% CI)	*P*-value
NDVI 1 year before exam						
$NDVI_{5km}$	-6.25 (-9.73, -2.78)	<0.001	-3.44 (-6.43, -0.45)	0.024	-2.77 (-5.83, 0.28)	0.075
$NDVI_{2km}$	-7.02 (-10.69, -3.34)	<0.001	-3.46 (-6.66, -0.26)	0.034	-2.54 (-5.85, 0.75)	0.131
$NDVI_{1km}$	-6.68 (-10.37, -2.98)	<0.001	-3.09 (-6.31, 0.13)	0.060	-2.17 (-5.50, 1.14)	0.198
$NDVI_{500m}$	-5.67 (-9.33, -2.01)	0.002	-2.38 (-5.57, 0.81)	0.144	-1.49 (-4.76, 1.77)	0.370
$NDVI_{250m}$	-5.03 (-8.76, -1.29)	0.008	-1.77 (-5.03, 1.48)	0.285	-0.84 (-4.18, 2.49)	0.618
NDVI 2 years before exam						
$NDVI_{5km}$	-7.07 (-10.56, -3.58)	<0.001	-4.25 (-7.25, -1.25)	0.005	-3.64 (-6.70, -0.57)	0.019
$NDVI_{2km}$	-7.44 (-11.07, -3.81)	<0.001	-3.91 (-7.05, -0.78)	0.014	-3.18 (-6.24, 0.04)	0.053
$NDVI_{1km}$	-7.07 (-10.71, -3.42)	<0.001	-3.57 (-6.72, -0.41)	0.026	-2.83 (-6.08, 0.41)	0.087
$NDVI_{500m}$	-6.13 (-9.74, -2.51)	<0.001	-2.90 (-6.03, 0.22)	0.068	-2.17 (-5.38, 1.02)	0.182
$NDVI_{250m}$	-5.41 (-9.07, -1.74)	0.004	-2.28 (-5.46, 0.89)	0.158	-1.51 (-4.77, 1.73)	0.361
NDVI 3 years before exam						
$NDVI_{5km}$	-6.92 (-10.41, -3.43)	<0.001	-4.12 (-7.12, -1.12)	0.007	-3.51 (-6.57, -0.44)	0.024
$NDVI_{2km}$	-7.30 (-10.94, -3.66)	<0.001	-3.74 (-6.89, -0.60)	0.019	-3.00 (-6.24, 0.23)	0.069
$NDVI_{1km}$	-6.93 (-10.58, -3.27)	<0.001	-3.39 (-6.56, -0.23)	0.003	-2.65 (-5.91, 0.60)	0.110
$NDVI_{500m}$	-6.04 (-9.68, -2.41)	0.001	-2.78 (-5.92, 0.36)	0.082	-2.04 (-5.27, 1.17)	0.213
$NDVI_{250m}$	-5.27 (-8.95, -1.59)	0.004	-2.14 (-5.33, 1.04)	0.187	-1.36 (-4.62, 1.89)	0.412

NDVI: Normalized Difference Vegetation Index. β represents changes in GrimAA per 0.1 increment of NDVI.
Model 1: adjusted for age, sex, race, and field center; Model 2: adjusted for Model 1 covariates + individual behavior and SES (education years, smoking, marital status, income, physical activity, and BMI); Model 3: adjusted for Model 2 covariates + neighborhood deprivation score.

Supplementary Table 8. Associations of long-term exposure to residential surrounding greenness (1985-2006; Y0-Y20) with PC-based GrimAA and PC-based PhenoAA (2005-2006; Y20)

	PC-based GrimAge		PC-based PhenoAge	
	β (95% CI)	*P*-value	β (95% CI)	*P*-value
Having parks within 5km (yes vs. no)	-0.31 (-0.64, 0.02)	0.069	-0.54 (-1.33, 0.24)	0.179
NDVI 5km, before 1yr	-1.86 (-3.77, 0.04)	0.055	-1.04 (-5.47, 3.38)	0.642
NDVI 5km, before 2yr	-2.36 (-4.46, -0.26)	0.027	-2.51 (-7.07, 2.04)	0.279
NDVI 5km, before 3yr	-2.36 (-4.46, -0.27)	0.026	-2.52 (-7.07, 2.02)	0.277

NDVI: Normalized Difference Vegetation Index. β for NDVI represents changes in PC-based EAA per 0.1 increment of NDVI. Models were adjusted for age, sex, race, education years, smoking, marital status, income, physical activity, BMI, neighborhood deprivation score, and field center.

Supplementary Table 9. Sensitivity analyses for associations of long-term exposure to residential surrounding greenness (1985-2006; Y0-Y20) with EAA (2005-2006; Y20), additionally adjusted for cumulative smoking and leukocyte compositions.

	Model 3 + cumulative smoking		Model 3 + leukocyte compositions	
	beta (95% CI)	P-value	beta (95% CI)	P-value
GrimAA				
Having parks within 5km (yes vs. no)	-0.35 (-0.80, 0.08)	0.110	-0.35 (-0.78, 0.08)	0.115
NDVI 5km, before 1yr	-1.88 (-3.93, 0.16)	0.071	-1.78 (-3.86, 0.29)	0.093
NDVI 5km, before 2yr	-2.42 (-4.59, -0.24)	0.029	-2.21 (-4.41, -0.01)	0.048
NDVI 5km, before 3yr	-2.40 (-4.56, -0.24)	0.029	-2.17 (-4.35, 0.01)	0.051
PhenoAA				
Having parks within 5km (yes vs. no)	-0.36 (-1.12, 0.38)	0.338	-0.07 (-0.76, 0.62)	0.842
NDVI 5km, before 1yr	-3.66 (-7.77, 0.44)	0.080	-2.22 (-6.03, 1.57)	0.251
NDVI 5km, before 2yr	-4.52 (-8.73, -0.31)	0.035	-3.12 (-7.06, 0.81)	0.120
NDVI 5km, before 3yr	-4.50 (-8.68, -0.31)	0.035	-3.04 (-6.96, 0.87)	0.127
DunedinPACE				
Having parks within 5km (yes vs. no)	-0.00 (-0.01, 0.01)	0.961	0.00 (-0.01, 0.01)	0.815
NDVI 5km, before 1yr	0.02 (-0.04, 0.09)	0.499	0.02 (-0.03, 0.09)	0.398
NDVI 5km, before 2yr	0.01 (-0.06, 0.07)	0.884	0.01 (-0.05, 0.07)	0.773
NDVI 5km, before 3yr	0.01 (-0.06, 0.07)	0.847	0.01 (-0.05, 0.08)	0.718

Model 3 included age, sex, race, education years, smoking, marital status, income, physical activity, BMI, neighborhood deprivation score, and field center as covariates.
